# Complex Hernia Presentation: A Case Study of Concurrent Spigelian and Pantaloon Hernias in a 49-Year-Old Female

**DOI:** 10.7759/cureus.60070

**Published:** 2024-05-10

**Authors:** Pratik S Navandhar, Pankaj Gharde, Raju K Shinde, Tushar Nagtode, Bhagyesh Sapkale, Varun Kulkarni

**Affiliations:** 1 General Surgery, Jawaharlal Nehru Medical College, Datta Meghe Institute of Higher Education and Research, Wardha, IND; 2 Medicine, Jawaharlal Nehru Medical College, Datta Meghe Institute of Higher Education and Research, Wardha, IND

**Keywords:** computed tomography, physical examination, lichtenstein procedure, surgical repair, concurrent hernias, case study, pantaloon hernia, spigelian hernia, inguinal hernia, hernia

## Abstract

A 49-year-old woman with a complicated hernia presentation, including direct and indirect inguinal hernias, Spigelian hernias, and Pantaloon hernias, is presented in the case report. The diagnosis was verified by a comprehensive physical examination and imaging, which resulted in a Lichtenstein operation for repair. The surgical procedure for hernia comprised of painstaking dissection, reduction of the hernia sac, and implantation of a prosthetic mesh. The instance emphasizes the value of individualized treatment programs and draws attention to the intricate anatomical details of hernia surgery. Analyzing situations that are similar to one another highlights the necessity of customized strategies to improve patient outcomes.

## Introduction

When tissue, such as a portion of the intestine, pokes through a weak area in the abdominal muscles, it can cause an inguinal hernia [1]. The ensuing bulge may hurt, particularly if you cough, stoop, or pick up something heavy. Nonetheless, a lot of hernias are painless. A direct inguinal canal can't reach the scrotum [2]. When a section of the abdomen known as the inguinal rings fails to close during infancy, an indirect inguinal hernia can form that extends to the scrotum [1,2]. Increasing intra-abdominal pressure during the Valsalva maneuver can make herniated tissue more noticeable when palpated, which is why it is used to diagnose inguinal hernias [3]. This strategy assists physicians in identifying hernias through physical examination, aiding diagnosis and therapy planning. In women, an indirect inguinal hernia can form if reproductive organs or the small intestine moves into the groin area because of a weakening in the abdominal muscles [1]. Adults are more prone to develop a direct inguinal hernia, which is brought on by the weakening of the abdominal muscles over time [1,2]. A Spigelian hernia is an uncommon ventral hernia characterized by the protrusion of the peritoneum or abdominal contents through a defect in the Spigelian fascia [4]. A combination of two contiguous hernia sacs in the femoral or inguinal areas on the same side is called a pantaloon hernia [5]. As a result, the inferior epigastric arteries have sacs on both sides [5]. This case report aims to highlight the complexity of hernia presentations and underscore the necessity for tailored treatment strategies to optimize patient outcomes while also contributing to understanding variations in surgical approaches and outcomes through comparisons with similar cases.

## Case presentation

A 49-year-old female presented to the Acharya Vinoba Bhave Rural Hospital (AVBRH), Wardha, India, with a complaint of swelling in the left side of her abdomen that had been progressively increasing over the past month. During a physical examination, the healthcare provider assesses for the presence of inguinal hernias, which are protrusions of abdominal contents through weak spots or defects in the inguinal canal. The examination typically involves direct and indirect methods to differentiate between these hernias. The patient was asked to stand and cough in a direct inguinal hernia examination. Then, a Valsalva maneuver was performed (holding the breath and bearing down as if having a bowel movement). The examiner palpated the inguinal canal and the area just above the inguinal ligament. The protrusion appeared medial to the inferior epigastric vessels, and this region felt bulging. The hernia was reducible and can be pushed back into the abdominal cavity. Additionally, the examiner noted tenderness or discomfort upon palpation.

**Figure 1:**
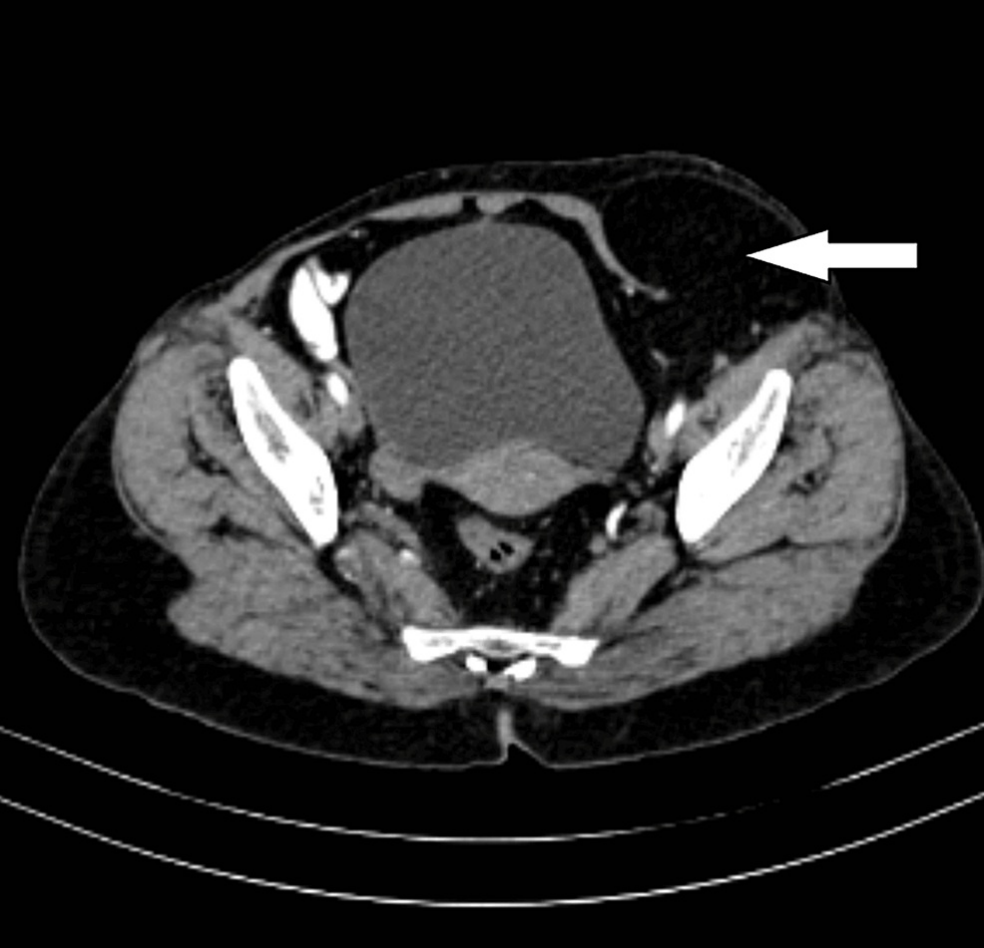
Computed tomography showing the outpouching of bowel loops The white arrow indicates outpouching of the bowel loops through the anterior abdominal wall

The examination method is similar for indirect inguinal hernias but involves a different palpation technique. The examination started by placing a finger in the inguinal canal and asking the patient to cough, then performing the Valsalva maneuver. The examiner felt a bulge or swelling lateral to the inferior epigastric vessels as the herniated contents protruded through the internal inguinal ring in indirect inguinal hernias. Overall, a thorough physical examination was done using both direct and indirect techniques, which is essential to accurately diagnose inguinal hernias and determine the appropriate course of treatment. On physical examination, both indirect and direct inguinal hernias were palpable, and further imaging via computed tomography (CT) revealed the presence of a Spigelian hernia on the left side, as shown in Figure 1.

**Figure 2:**
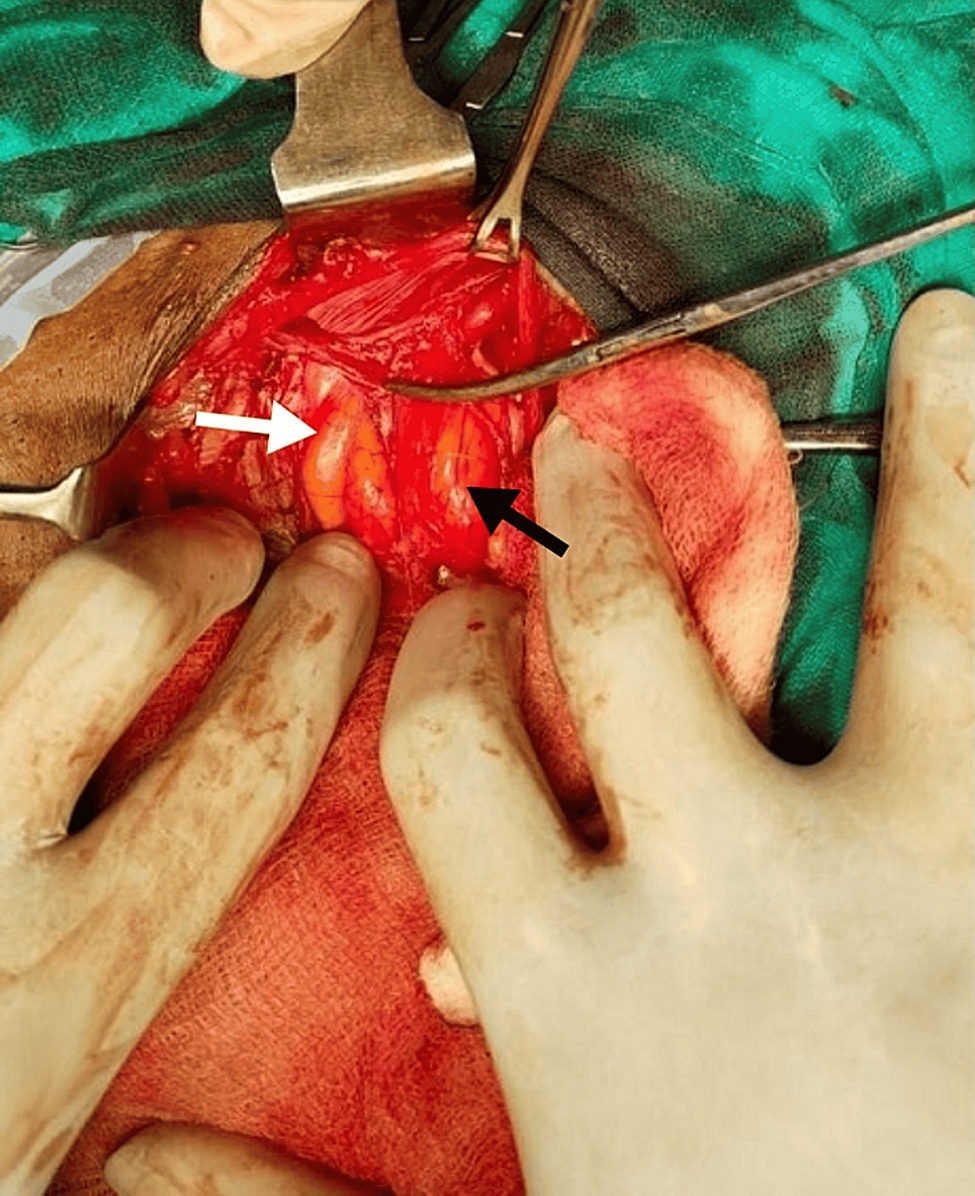
Intra-operative findings showing Pantaloon hernia A protrusion medial to the inferior epigastric arteries is indicated by a white arrow (direct inguinal hernia) An indirect inguinal hernia can be seen as a bulge lateral to the inferior epigastric vessels, as shown by the black arrow The inferior epigastric vessels are shown by the artery forceps' tip

During the Lichtenstein surgical procedure, a standard infra umbilical incision was made. Intraoperatively, the outpouching was observed laterally and medially to the inferior epigastric vessels, consistent with the diagnosis of concurrent indirect and direct inguinal hernias. Additionally, bowel loops were identified as the contents of the hernial sac, indicative of a Pantaloon hernia, as represented in Figure 2.

Given the complexity of the hernia presentation, the Lichtenstein procedure was chosen for hernia repair. A 49-year-old female underwent hernia repair surgery, which involved several vital steps to ensure a successful outcome. Initially, she was prepared for the procedure by being comfortably positioned on the operating table, and the surgical site was meticulously cleaned and draped to reduce the risk of infection. An incision was then made over the hernia site, allowing access to the hernia sac and surrounding tissues. The sac and its contents were carefully dissected and returned to the abdominal cavity. Multiple defects were addressed if necessary, such as in the case of a Pantaloon hernia. Following this, a prosthetic mesh made of polypropylene was placed over the hernia defect to reinforce the weakened abdominal wall and prevent recurrence. The mesh was securely fixed using non-absorbable sutures or staples, and the incision was closed in layers. In our case, the patient receives postoperative care focused on pain management, wound care, and early mobilization to support her recovery. The procedure was performed successfully, resulting in the complete closure of all hernial defects and restoring the integrity of the abdominal wall. Regular follow-up visits were scheduled to monitor her progress and discuss long-term outcomes, including the risk of hernia recurrence. The patient was closely monitored in the immediate postoperative period to ensure stabilization and proper wound healing. Pain management was administered as needed, and the wound site was regularly inspected for signs of infection or complications. Additionally, the patient was encouraged to begin early mobilization under healthcare professionals' guidance to prevent postoperative complications such as deep vein thrombosis or muscle weakness. The patient received counseling on lifestyle modifications to minimize the risk of hernia recurrence, including avoiding heavy lifting, maintaining a healthy weight, and engaging in appropriate physical activity. Dietary recommendations were provided to promote bowel regularity and prevent constipation, which can increase intra-abdominal pressure and strain the repaired hernia site. Long-term follow-up appointments were scheduled to monitor the patient's condition and evaluate the success of the surgical repair in preventing hernia recurrence. Imaging studies such as ultrasound and CT scans were ordered with concerns about the integrity of the abdominal wall or signs of hernia recurrence. The patient's two-year follow-up care involved a multidisciplinary approach to optimize her recovery and long-term outcomes, including collaboration between surgeons, primary care providers, and other healthcare professionals. Through careful monitoring and tailored interventions, the goal was to ensure the patient's well-being and minimize the risk of complications associated with hernia repair surgery. The timeline of events in the case is depicted in Table 1.

**Table 1:** Timeline of events AVBRH: Acharya Vinoba Bhave Rural Hospital; CT: Computed tomography Pantaloon hernia: Concurrent indirect and direct inguinal hernias with bowel loops Lichtenstein procedure: A type of hernia repair surgery that involves the placement of a mesh to reinforce the abdominal wall Incision: A surgical cut or wound made to gain access to the body's interior Post-operative: Referring to the period immediately following surgery Mesh: A synthetic material used in hernia repair to reinforce the weakened area of the abdominal wall Defects: Weak spots or openings in the abdominal wall Wound care: Treatment administered to a wound to promote healing and prevent infection Follow-up visits: Subsequent appointments scheduled after surgery to monitor progress and address any concerns Recurrence: The reappearance of a medical condition after it has been treated or resolved

Time	Event
One month before AVBRH visit	The patient experienced progressive swelling in the left abdomen.
AVBRH visit	The patient presented with a complaint of abdominal swelling.
	A physical examination was performed, including an evaluation of the inguinal hernias.
	Direct and indirect inguinal hernia examinations were conducted.
	Both direct and indirect hernias were initially suspected.
	CT scan revealed the presence of a Spigelian hernia.
Surgery	The patient underwent general anesthesia.
	Standard infra umbilical incision made.
	Intraoperative findings revealed concurrent indirect and direct inguinal hernias with bowel loops (Pantaloon hernia).
	Lichtenstein procedure was chosen for repair.
	Surgical site prepared and incision made.
	The hernia sac and contents were dissected and returned to the abdominal cavity.
	Defects were addressed, and the prosthetic mesh was placed for reinforcement.
	The mesh was secured, and the incision was closed in layers.
Post-surgery	The patient received post-operative care, including pain management, wound care, and early mobilization.
	Follow-up visits were scheduled to monitor progress and discuss long-term outcomes.
	The patient was advised to maintain a healthy lifestyle to minimize recurrence risk.
Surgery outcome	The successful procedure resulted in the complete closure of hernial defects and restored abdominal wall integrity.

## Discussion

Meaningful information on the therapy, clinical presentation, and outcomes in different age groups can be obtained by contrasting the cases of our 49-year-old patient with a Spigelian hernia and a 60-year-old woman with a similar diagnosis as Alshihmani et al. reported in a case [6]. First, Spigelian hernias are comparatively uncommon compared to others, which is the case in both circumstances. The 49-year-old female patient at AVBRH underwent laparoscopic repair, a minimally invasive procedure linked to a quicker recovery and less discomfort following surgery than open surgery. In contrast, an oblique incision was made across the edema during the exploration procedure on the 60-year-old female patient, who underwent mesh hernioplasty and anatomical repair afterward [6]. This case implies that various factors may influence the selected surgical method, including patient age, comorbidities, and physician preference.

In the case of a 69-year-old woman reported by Tatara et al., iatrogenic damage played a role in the development of a hernia, as the patient underwent laparoscopic ventral hernia repair and later acquired a Spigelian hernia [7]. After the initial laparoscopic repair of a ventral hernia, the abdominal wall's muscle layer ruptured, unintentionally resulting in the creation of a Spigelian hernia at the mesh's edge. This case emphasizes the possible dangers of surgical treatments and the significance of giving anatomical structures due consideration when performing hernia repair techniques [7]. In our case of a 49-year-old female, the patient underwent open repair of the Spigelian hernia, likely necessitated by the complexity of concurrently addressing both the Spigelian and Pantaloon hernias.

The mentioned case of a 49-year-old female with a Spigelian hernia accompanied by a pantaloon hernia, as well as a 46-year-old male's case documented by Wani with a "Double Pantaloon hernia," pose distinct complexities in both diagnosing and treating inguinal hernias [8]. These cases highlight the intricacies and variations in hernia presentations, necessitating careful evaluation and tailored surgical approaches for effective management. While the Spigelian hernia involves protrusion through the Spigelian fascia, the double Pantaloon hernia is characterized by a rare combination of two direct and three indirect inguinal hernias, complicated by an anomalous inferior epigastric artery [8]. Surgical management in both cases requires meticulous exploration and individual ligation of hernia sacs to prevent recurrence. These cases highlight the anatomical complexities, surgical approaches, and implications for patient outcomes, emphasizing the importance of thorough examination and tailored interventions to address each patient's needs.

## Conclusions

In conclusion, the case study of a 49-year-old woman with a Pantaloon hernia, in addition to contemporaneous direct and indirect inguinal hernias, highlights the variety and complexity of hernia presentations. Such instances require careful surgical skills, a thorough understanding of the anatomy of hernias, and customized treatment plans. Moreover, the comparative analysis with similar cases underscores the variety of surgical alternatives accessible and underscores the significance of customizing procedures to suit the particular case of every patient. This case report serves as a reminder of the anatomical complexity of hernia repair and the need for careful assessment and individualized treatment planning for the best possible results.
